# Primary Metabolism of Chickpea Is the Initial Target of Wound Inducing Early Sensed *Fusarium oxysporum* f. sp. *ciceri* Race I

**DOI:** 10.1371/journal.pone.0009030

**Published:** 2010-02-03

**Authors:** Sumanti Gupta, Dipankar Chakraborti, Anindita Sengupta, Debabrata Basu, Sampa Das

**Affiliations:** 1 Plant Molecular and Cellular Genetics Section, Bose Institute, Kolkata, India; 2 Botany Department, Bose Institute, Kolkata, India; CNRS UMR 8079/Université Paris-Sud, France

## Abstract

**Background:**

Biotrophic interaction between host and pathogen induces generation of reactive oxygen species that leads to programmed cell death of the host tissue specifically encompassing the site of infection conferring resistance to the host. However, in the present study, biotrophic relationship between *Fusarium oxysporum* and chickpea provided some novel insights into the classical concepts of defense signaling and disease perception where ROS (reactive oxygen species) generation followed by hypersensitive responses determined the magnitude of susceptibility or resistant potentiality of the host.

**Methodology/Principal Findings:**

Microscopic observations detected wound mediated *in planta* pathogenic establishment and its gradual progression within the host vascular tissue. cDNA-AFLP showed differential expression of many defense responsive elements. Real time expression profiling also validated the early recognition of the wound inducing pathogen by the host. The interplay between fungus and host activated changes in primary metabolism, which generated defense signals in the form of sugar molecules for combating pathogenic encounter.

**Conclusions/Significance:**

The present study showed the limitations of hypersensitive response mediated resistance, especially when foreign encounters involved the food production as well as the translocation machinery of the host. It was also predicted from the obtained results that hypersensitivity and active species generation failed to impart host defense in compatible interaction between chickpea and *Fusarium*. On the contrary, the defense related gene(s) played a critical role in conferring natural resistance to the resistant host. Thus, this study suggests that natural selection is the decisive factor for selecting and segregating out the suitable type of defense mechanism to be undertaken by the host without disturbing its normal metabolism, which could deviate from the known classical defense mechanisms.

## Introduction

Resistance in many plant-pathogen interactions is associated with multifaceted defense systems. The individual components of such systems include hypersensitive responses, chemical weapons like phytoalexins and hydrolytic enzymes, and structural barriers like lignin and hydroxyproline rich cell wall proteins [1]. Proper recognition and judicious regulation of defense responses is essential for host plants, as these responses often have small (but measurable) deleterious effects on plant growth and metabolism [2]. Fungal pathogens deploy different strategies to escape host surveillance and establish themselves within the host depending on their nutritional requirements [3]. Necrotrophic pathogens derive their nutrition from the dead and decomposed material of the host. Biotrophic fungi diplomatically adapt themselves to the host, derive nutritional prerequisites and then categorically overpower them. The hyphae of biotrophs grow both inter- and intracellularly, and become encompassed by the host plasma membrane. The causal agents of rusts and powdery mildew disease develop specialized nutrition sucking devices named ‘haustoria’ [4]. These carbohydrate and protein interfaces between the host plasma membrane and penetrating hyphae facilitate the constant exchange of signals and nutrients between the interacting partners [5]. This intimate interface ultimately becomes the decisive factor for the outcome of the interaction, whether it is fatal or conducive for both the host and the intruder [6].

The molecular bases for the recognition of biotrophs by plants outside the purview of gene-for-gene systems are still elusive. Plants usually recognize pathogen-associated molecular patterns (PAMPs) in the form of chitin, glucan fragments or pathogen recognition receptor (PRR) proteins. Sometimes pathogen-mediated degraded cell wall polysaccharides of plant origin also serve as elicitors. After pathogen recognition, a multitude of plant resistance-associated reactions are initiated, such as ion fluxes across plant membranes, the generation of reactive oxygen species (ROS), phosphorylation of specific proteins, activation of cell wall strengthening enzymes, transcriptional activation of several defense related genes, induction of phytoalexins, localized cell death at infection sites (HR response), and induction of systemic acquired resistance in distal plant organs [7]. Gene-for-gene recognition of the pathogen corresponding *R-avr* of host and pathogen also triggers ROS generation followed by programmed cell death (PCD) at the site of infection [2]. In the case of obligate biotrophs, R gene-mediated defenses are reported to trigger salicylic acid (SA)-dependent defense responses downstream and thus restrict the pathogenic invasion. Conversely, in the case of necrotrophs, programmed cell death supports the growth of the pathogen. As a result, jasmonic acid and ethylene (JA/ET)-dependent signaling is reported to be operational in the case of necrotrophs [8].

*Fusarium oxysporum* f. sp. *ciceri* is an important obligate biotroph that causes vascular wilt disease of chickpea. Chickpea (*Cicer arietinum* L.) is an important source of plant-derived edible protein. It occupies the third position in the list of important pulse crops of the world [9]. This most important pulse crop of India and its adjoining countries account for 90% of the total world production [10]. North and Central America produce about 5% of the world production [10]. Since chickpea is affordable to the general population it is widely used as a substitute for animal protein. But the yield of this crop is severely affected by *F. oxysporum* f. sp. *ciceri* attack. Annual losses account for 10–15% of the total yield, and this sometimes escalate to total loss under specific conditions [11]. This seed or soil borne pathogen has two different pathotypes. The yellowing pathotype produces foliar yellowing followed by vascular discoloration, while the more devastating wilt-causing pathotype induces severe and fast chlorosis, flaccidity and vascular discoloration [11]. The fungus colonizes the xylem vessels and thus prevents the translocation of water and nutrients, resulting in wilting [12]. Eight pathogenic races (0, 1, 1B/C, 2–6) of this monophyletic fungus are reported, amongst which races 0 and 1B/C induce yellowing while the rest cause wilting. Race 1, reported to have wide geographic distribution, is widely used by the scientific community to investigate plant-pathogen interactions [13].

*Fusarium* wilt is primarily managed by resistance breeding programs. But pathogenic variability and mutability leading to breakdown of naturally selected resistance are the main hurdles for plant breeders [14]. Marker-assisted gene mapping studies have been done by many research groups [15]. Post-pathogenic invasion related biochemical analyses have also been performed by many scientists [14], [12], [16], which suggests that the resistance against *F. oxysporum* f. sp. *ciceri* in chickpea is not governed by the classical SA dependent defense responses operational in traditional plant-biotrophic encounters. These studies emphasize the presence of some unconventional defense mechanism in this particular plant/pathogen interaction [17]. Unfortunately, researchers have not yet been able to provide satisfactory explanations for *in planta* pathogenic establishment and the corresponding plant reactions. Hence, this particular pathogenic invasion and its resultant host defense warrants extensive additional investigation.

To address this problem, we reported some differentially expressed expression sequenced tags (ESTs) from a case study of the *Fusarium*-chickpea pathosystem [18]. Our report suggested early recognition of the biotroph by the host. As a result, cascades of signaling molecules were generated that imparted downstream host defenses. In our present study, to understand how pathogenic entry is sensed within the host, we sought to identify the initial targets of the intruders and to determine how the plant reacts to the foreign invaders with its team of molecular warriors.

## Results

### Manifestation of Fungal Attack

**Figure 1:**
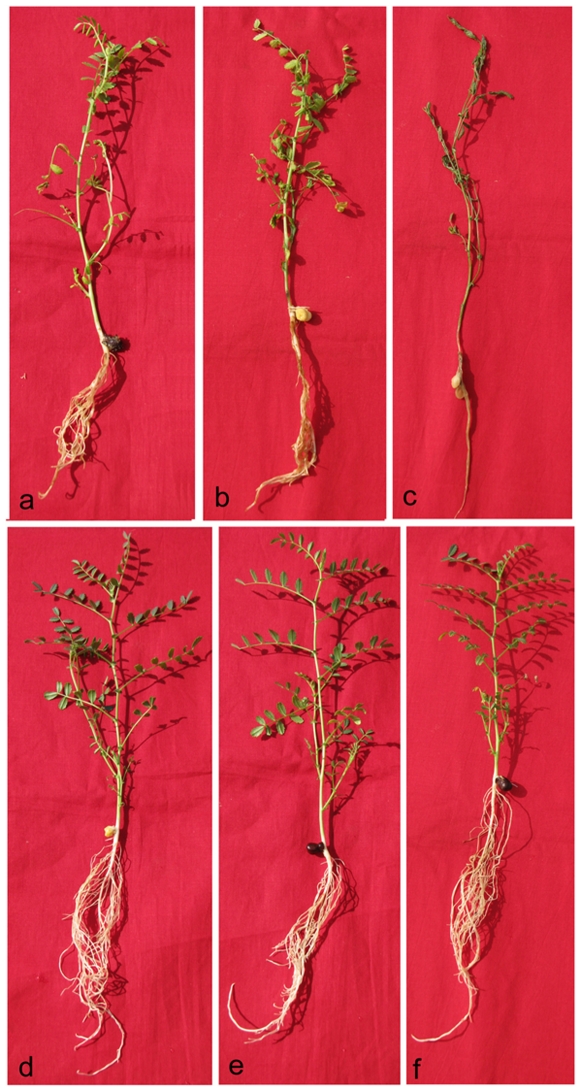
Phenotypical changes of chickpea plants upon *Fusarium oxysporum* f.sp. *ciceri* (Race 1) attack. Infected JG62 plants at 4DPI (a), 8DPI (b) and 12DPI(c). Infected WR315 plants at 4DPI (d), 8DPI (e) and 12DPI (f).

The initial symptoms of pathogenic infection were detected at four days post inoculation [DPI] in wilt-susceptible JG62 plants. Yellowing of rootlets, chlorosis of basal leaflets and slight drooping of lower branches were visible [Figure 1a]. The symptoms showed more prominence at 8 DPI with distinct browning of root zones (probably indicating the pathogenic entry points) [Figure 1b], retardation of root growth and branching accompanied by chlorosis of the upper branches. At 12 DPI the symptoms were further intensified. Root growth and branching were drastically affected; root browning to blackening due to extensive phenolic deposition occurred; chlorosis and drooping of the entire plant marked the onset of wilt [Figure 1c]. At 18–20 DPI about 90% of the susceptible plants wilted. However, the resistant WR315 plants, except for a slight yellowing of the roots, showed normal branching even at 15 DPI [Figure 1d, 1e, 1f]. The control plants of both wilt-susceptible JG62 and wilt-resistant WR315 showed normal root growth, indicating that the changes in the infected plant samples were the consequences of pathogen attack.

### Establishment of the Pathogen within the Host

**Figure 2:**
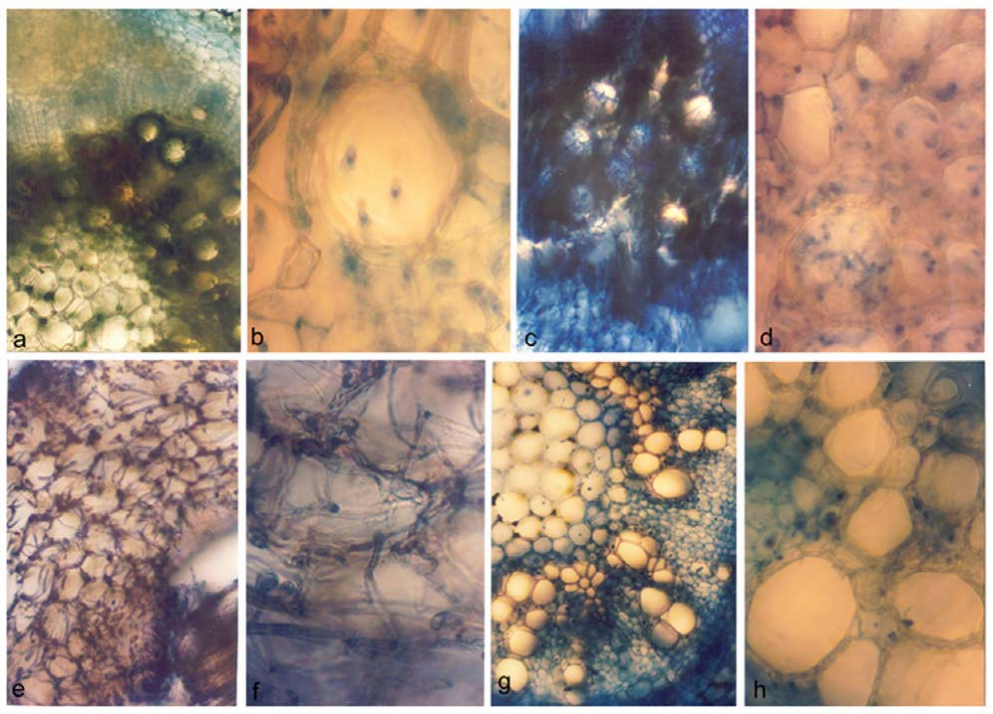
Sectional views of infected roots of chickpea plants stained with Trypan blue and lactophenol. Root section of infected JG62 plants at 4DPI (a and b), 8DPI (c and d) and 12DPI (e and f). Bars represent 10 µm.

Serial sectioning of infected roots of both the varieties was done every 24 hours post-inoculation to determine the onset of pathogen colonization in the xylem vessels. Trypan blue and lactophenol staining of the infected sections confirmed the presence of the fungus in the xylem vessels of wilt-susceptible JG62 at 4 DPI [Figure 2a, 2b]. Phenolic deposition was evident at a small number of vessels although tissue disintegration was not pronounced. Extensive fungal ramification coupled with tissue disintegration and heavy phenolic deposition was found at 8 DPI [Figure 2c, 2d]. Approximately 75% of the xylem vessels exhibited a clogged appearance. At 12 DPI, fungal invasion and subsequent phenolic deposition was found to totally obstruct the xylem vessels of JG62. Also, vascular and ground tissue disintegration occurred [Figure 2e, 2f]. Serial sectioning of infected JG62 roots was not possible after 12 DPI due to total loss of normal root architecture. However, in the wilt-resistant WR315 plants no signs of vascular clogging were seen even after 12 DPI [Figure 2g, 2h]. The control roots of both the varieties showed normal anatomical profiles.

**Figure 3:**
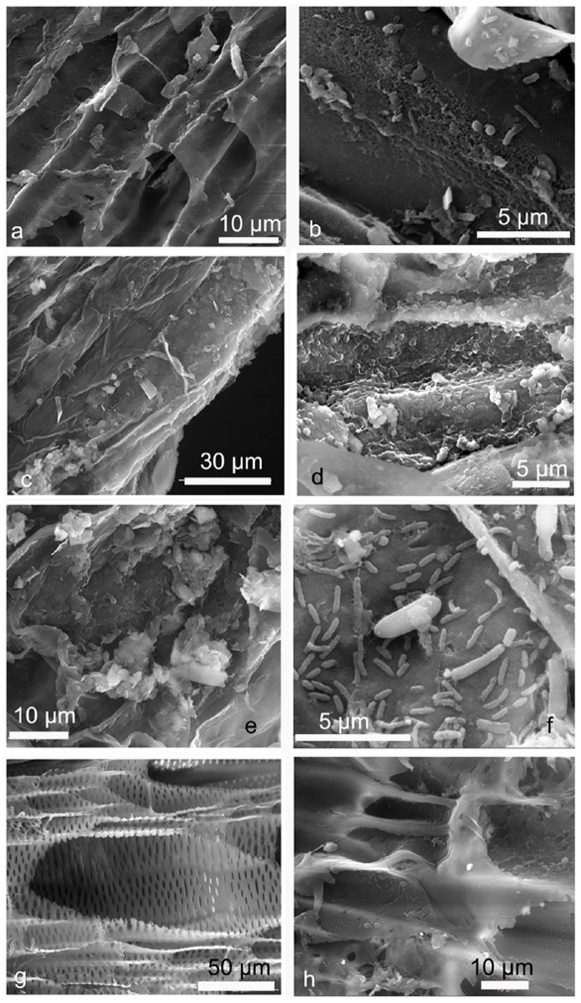
Scanning electron micrographs of infected roots of chickpea plants. Root section of infected JG62 plants at 4 DPI, 8 DPI, 12 DPI showing tissue disintegration (a), (c), (e) and conidia (b), (d), (f), respectively. Root section of infected WR315 plants at 15DPI showing xylem vessels (g) and tissue damage with conidia at 28DPI (h).

Light microscopic results were further confirmed by scanning electron microscopy [SEM]. Fungal microspores were visible at the xylem tissue interior of infected JG62 plants at 4 DPI [Figure 3b]. Onset of tissue damage was also observed (Figure 3a). At 8 DPI, a large number of spores were found [Figure 3d]. The vascular tissue damage was more pronounced [Figure 3c]. At 12 DPI, the fungal spores not only increased in number but were also found at different divisional stages, with macroconidia in chains being quite characteristic [Figure 3f]. The original tissue architecture was almost abolished [Figure 3e]. On the other hand, infected root sections of WR315 showed no anomaly even after 15 days of fungal entry [Figure 3g]. The fungal spores were detected at xylem vessels after 22–24 DPI. Some amount of fungal colonization with slight tissue disintegration was visible at 28 DPI [Figure 3h], but fungal spore divisions were not detected.

### *F. oxysporum* f. sp. *ciceri* Ra*ce1* (Foc Race 1) Mediated Changes in Host Transcription

**Figure 4:**
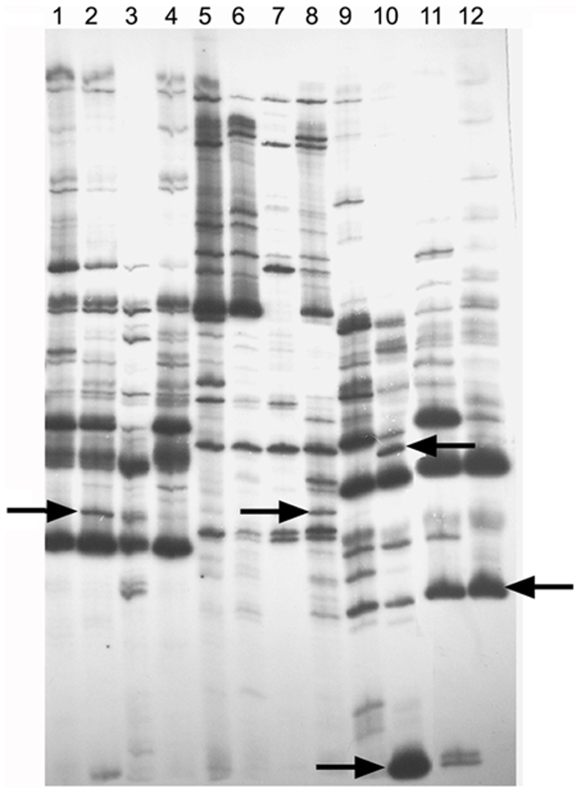
cDNA-AFLP gel profile of non-infected and infected JG62 and WR315 plant samples using different primers. Lanes 1, 5, 9 non-infected JG62; lanes 3,7,11 non-infected WR315; lanes 2, 6 and 10 infected JG62 and lanes 4, 8, 12 infected WR315. Primer combinations used were lanes 1 to 4, E-AGC/M-CAC; lanes 5 to 8, E-AGC/M-CAG and lanes 9 to 12 E-AGC/M-CAT. Arrows indicate some of the bands selected for further analysis.

cDNA-AFLP profiling was performed in chickpea for a comprehensive analysis of host cell responses generated prior to fungal establishment within the host. The differential transcript profiling generated an output of 1489 differential gene fragments. Among these differential gene fragments, 25% were detected due to fungal attack [Figure 4]. Some were over-expressed in the resistant variety, some in the susceptible variety and some were unique to a particular variety while being completely suppressed in its counterpart [Table S1]. All the distinctly upregulated, downregulated and uniquely expressed transcripts [ranging 50–400 bp] were eluted, sequenced and submitted to the EST database of Genbank. Out of 87 distinct gene fragments, 25 were found to be repetitive sequences and were excluded from the EST list [Table S1]. Among the differential ESTs obtained, many shared similarity with known genes, some with proteins of unknown function and the rest with un-annotated clones. The results obtained through cDNA-AFLP were further validated using qPCR where the relative expression levels of many of these characteristic gene fragments were calculated [Table S2].

### Early Plant Responses to Foc Race 1

**Figure 5:**
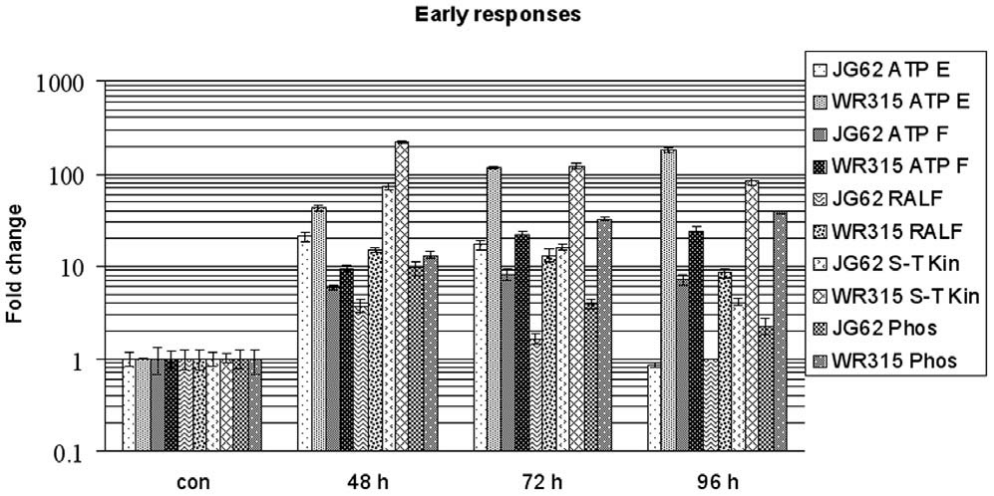
Relative expression of early defense response genes. Expression of ATPase E and F subunit, rapid alkalinization factor, serine threonine kinase and phopholipase C at 48, 72 and 96 hours post fungal induction in JG62 and WR315 plants. Error bars represent standard error (n = 3).

The expression profiles showed an early induction of several defense responsive genes in both varieties prior to fungal establishment in host vascular tissue, although the level or trend of expression was not the same in the two varieties. All the expression data were calculated in terms of fold-change relative to calibrator control samples [Table S2, Figure 5]. The expression of ATPase subunit E transcript showed an opposite trend in the two infected varieties. At 2 DPI, the resistant variety showed an almost two-fold increase in ATPase subunit E compared to the susceptible variety, and this further increased and exhibited the highest level of expression at 4 DPI. Conversely, the expression of ATPase subunit E transcript at 2 DPI in infected susceptible plants decreased at 3 DPI with a further sharp decline at 4 DPI. The expression of ATPase subunit F transcript showed similar trends in both the infected varieties. However, the resistant variety showed an increment of almost 1.5-fold at 2 DPI, 2.5-fold at 3 DPI and 3.5-fold at 4 DPI compared to the susceptible plants. Rapid alkalinization factor [RALF] related EST showed the highest degree of expression in resistant plants at 2 DPI, which gradually decreased with time, whereas its expression was almost 5-fold less in the susceptible variety compared to resistant plants at 2 DPI, and this further declined. ESTs of Serine/Threonine protein kinase and phospholipase C exhibited similar expression patterns in both infected samples. However, the levels showed significant elevation throughout in the resistant variety compared to the susceptible ones. The initial levels of phospholipase expression in the resistant variety at 2 DPI was approximately 1.3-fold higher than the susceptible one, and this further increased at 3 DPI and maintained this level even at 4 DPI.

### Pathogen-Induced Wounding and Stress in Host Plants

**Figure 6:**
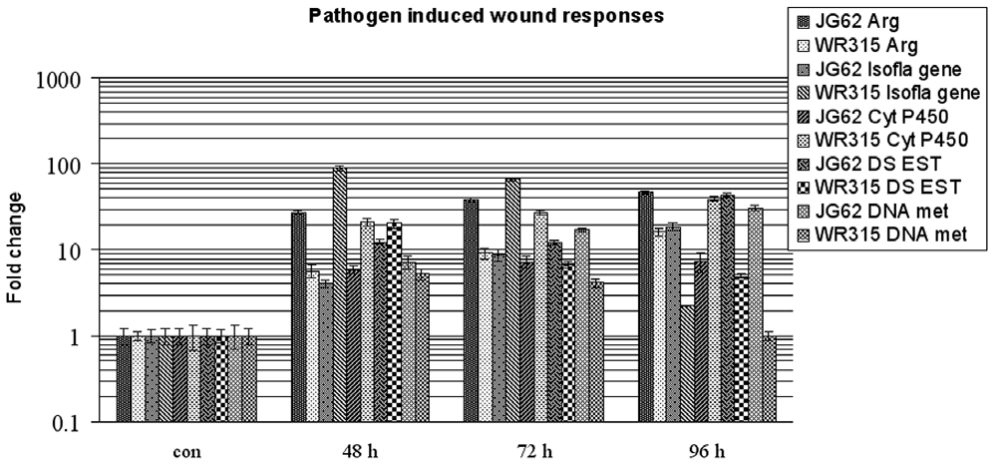
Relative expression of wound responsive genes. Expression of arginase, isoflavanoid biosynthetic gene, cytochrome P450 monoxygenase, drought stress ESTs and DNA methylation sensitive gene fragment at 48, 72 and 96 hours post fungal induction in JG62 and WR315 plants. Bars represent standard error (n = 3).

Similar expressional trends of transcripts of wound-responsive enzyme arginase in both the infected plant varieties emphasized wounding caused due to fungal penetration. The level of arginase was elevated concurrently with increasing time from 2 DPI to 4 DPI [Table S2, Figure 6]. However, the amount in the susceptible variety was much higher compared to the resistant one at any particular time point, suggesting more pronounced wounding in the susceptible variety. Isoflavanoid biosynthetic gene levels increased with time of infection in the susceptible variety, whereas the levels decreased with increasing time in the resistant variety. Interestingly, the isoflavanoid biosynthetic gene levels were approximately 22-fold and 8-fold higher in the resistant variety compared to the susceptible variety at 2 DPI and 3 DPI, respectively. But these levels showed a sharp fall at 4 DPI in the resistant variety and were found to be almost 9-fold lower than its susceptible counterpart. Cytochrome P450 transcript levels were found to be quite conserved throughout the pathogenic progression. However, the amounts were 3.5–5 fold higher in the resistant variety compared to the susceptible one. A DNA methylation-sensitive gene fragment was found to be overexpressed throughout in the susceptible variety. Besides, a drought stress-related EST that initially showed a 1.5-fold increase in the resistant variety compared to the susceptible one sharply declined at later time points, while the levels increased in the susceptible variety with increasing time.

### Changes in Primary Plant Metabolism

**Figure 7:**
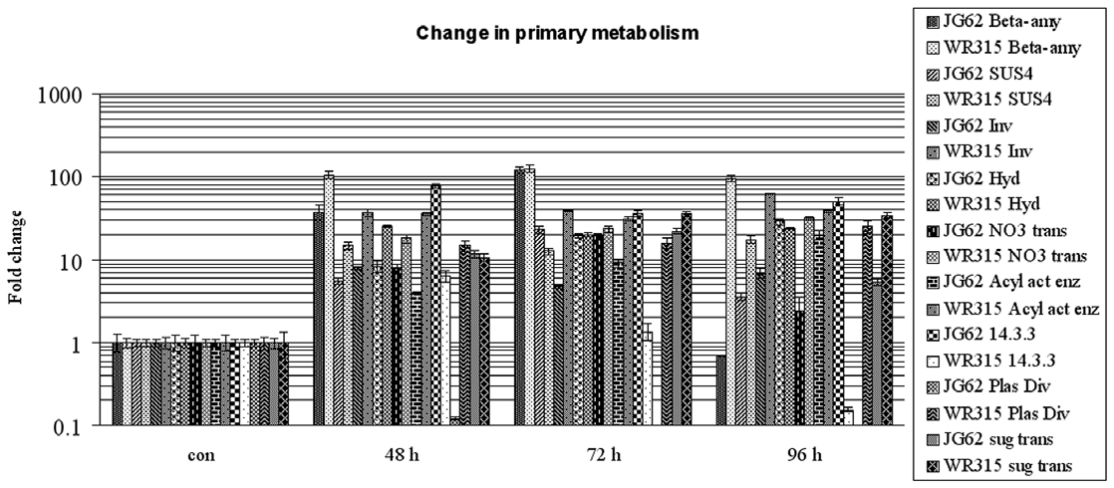
Relative expressions of genes related to primary metabolism. Expression of beta amylase, sucrose synthase, invertase, hydrolase, nitrate transporter, acyl activating enzyme, 14-3-3 related protein, plastid division regulator and sugar transporter at 48, 72 and 96 hours post fungal induction in JG62 and WR315 plants. Bars represent standard error (n = 3).

Pathogen-mediated alterations were evident from the expression of transcripts regulating source-sink ratios. Carbon stress probably due to higher energy consumption as a result of pathogen ingression was suggested by the high transcript levels of beta amylase, sucrose synthase and invertase found in resistant plants [Table S2, Figure 7]. Beta amylase levels were maintained in the resistant cultivar from 2 DPI–4 DPI, while the levels fell drastically after attaining a peak at 3 DPI in susceptible plants. Similar results were found for sucrose synthase in both resistant and susceptible varieties with the exception that the lower levels did not reach the basal value in the susceptible variety as found in the case of beta amylase. The expression of invertase was quite different from the previous two as a constant level was maintained in the susceptible variety, whereas the resistant variety showed a gradual increase in enzyme content with increasing time. The hydrolase transcript levels increased with infection progression in the susceptible variety, probably indicating pathogen-governed hydrolysis taking place within the host interior along with fungal ramification. Sugar transport was probably maintained during stressful periods as supported by the increment of sugar transporter ESTs at later periods of 4 DPI in resistant varieties. On the other hand, the transporter levels decreased after giving a peak at 3 DPI in susceptible varieties. The altered levels of nitrate transporters also suggest changes in nitrogen metabolism probably due to pathogenic attack or the result of carbon stress. Nitrate transporter expression increased over time in the resistant variety, whereas it showed a prominent decrease at 4 DPI in the susceptible variety. Transcripts of acyl activating enzyme levels increased concomitantly post-inoculation in the susceptible variety while they remained at distinctly higher levels from 2 DPI–4 DPI in resistant varieties. The gene expression of 14.3.3 was characteristically high from 2 DPI–4 DPI in susceptible varieties, suggesting some other significant role apart from mediating stress signals. The expression of this protein transcript was negligible in the resistant variety. On the contrary, expression of a plastid division regulator related EST was very much significant in resistant variety, whereas in susceptible variety the expression was almost beyond detection.

### Pathogens Induce Transcriptional Regulators and Structural Components

**Figure 8:**
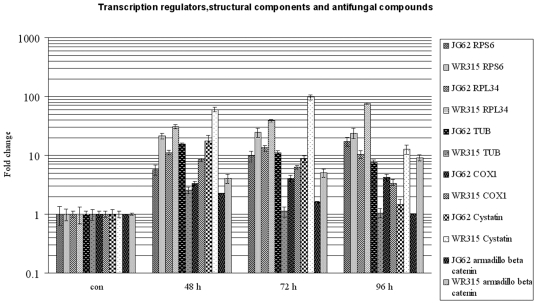
Relative expression of transcription regulators, structural and antifungal genes. Expression of Ribosomal protein RPS6 and RPL34, armadillo beta catenin repeat like protein, tubulin folding cofactor, cytochrome oxidase subunit 1 (COX1) and cystatin at 48, 72 and 96 hours post fungal induction in JG62 and WR315 plants. Bars represent standard error (n = 3).

ESTs showing similarity with ribosomal protein components like RPS6 and RPL34 showed varying degrees of expression in the two plant samples [Table S2, Figure 8]. RPS6 showed conserved expression in the resistant variety while its levels increased gradually with disease progression in the susceptible variety. RPL34 expression peaked at 4 DPI in resistant plants while susceptible plants showed minimum expression at 4 DPI. Armadillo beta catenin repeat protein transcript increased from 2 DPI–4 DPI in resistant plants while susceptible plants showed the opposite trend. Tubulin folding cofactor related EST showed prominent expression in susceptible plants whereas resistant plants exhibited negligible amounts. Level of transcripts of cytochrome oxidase subunit 1 (COX) showed reverse expressional trends of increment and decrement from 2 DPI–4 DPI in resistant and susceptible plants, respectively.

### Hosts Generates Antifungal Compounds

Antifungal compounds like cystatins related transcripts showed almost 3.5-fold induction at 2 DPI in resistant plants compared to susceptible plants, which further peaked at 3 DPI and then gradually decreased at 4 DPI. The susceptible plants showed a fair amount of expression at 2 DPI, which gradually decreased at 3 DPI and almost reached the basal level at 4 DPI [Table S2, Figure 8].

## Discussion

Advancement in agricultural research has drawn the scientific community towards understanding the host colonization mechanism of biotrophic fungi. Biotrophic fungi do not disturb host metabolism until they have fully equipped themselves to overpower the host defense machinery. This hypothesis is clearly supported by the *Arabidopsis*-*Perenospora* and *Arabidopsis*-*Erisiphe* case studies. In both cases, the pathogen does not alter the host's normal function until they are sheltered and have divided to produce second generation conidia [2]. In the present chickpea-*Fusarium* case study, results have provided some novel insights into the already established theories of plant-pathogen interactions and their downstream signals.

### Hypersensitive Response: Are Pathogens Always Restricted?

The obligate biotroph *F. oxysporum* penetrates the host through gaps between the root and root hairs, but it starts creating havoc only after entering the xylem vessels. *R*-gene mediated resistance usually accompanied by the accumulation of reactive oxygen species (ROS) culminates in the hypersensitive response (HR) and leads to programmed cell death (PCD) at the site of infection. The hypersensitive response is often associated with downstream SA signaling, especially in the case of biotrophs [8]. This sequential phenomenon is known to restrict the further invasion of the pathogen within the host [19]. Even though SA and *R*-gene mediated defense signaling have not yet been documented in the chickpea- *Fusarium* interaction, a hypersensitive response in the vascular tissue region is a reasonable assumption from the microscopic analyses done in the present study.

Fungal chitins, glucans and their degraded products function as PAMPs and trigger the hypersensitive response in host plants [6]. Degradation of the fungal chitins and glucans are governed by enzymes like chitinases and β, 1–3 glucanases of host origin [16]. Apart from degraded chitins and glucans, callose also acts as a positive regulator of the hypersensitive response [7]. Callose, a substrate of beta-1, 3 glucanase enzymes [20], deposits at the point of attempted penetration of the pathogen [21], [22] and functions as a host resistance factor. Whether the pathogen itself triggers the chitinase and glucanase activities or the PAMPs switch on the host defense is still debatable.

In the present study, the induction of these host enzymes was found to differ between the susceptible and resistant cultivars. The expression of chitinase and glucanases in the susceptible variety accentuated after 96 h of inoculation [23] when the pathogen already invaded the xylem vessel. Therefore, it is likely that the pathogen initially reprogrammed itself in such a fashion that its penetration was somehow aided by the host instead of being treated as a foreign invasion. The pathogen unveiled itself and started employing its pathogenic weapons against the host only after establishing itself within the xylem vessels. At this stage, the host chitinases and glucanases were induced. Induction of these enzymes resulted in the ROS-mediated hypersensitive response which often makes the plant susceptible instead of imparting resistance [24]. Moreover, SEM showed the accumulation of callose degradation products after fungal ramification inside the xylem of susceptible hosts, which may have aided in plugging the vessels resulting in blockage of upward translocation of mineral solutes. Callose encapsulation of haustoria occurring in incompatibility reactions between resistant hosts and pathogens prevent the pathogen's nutrient uptake. Conversely, in the case of compatible reactions between susceptible hosts and pathogens, β, 1–3 glucanase-induced callose degradation facilitates the absorption of nutrients by haustoria and promotes growth and sporulation [25]. Pathogenesis is characterized by the ability of the pathogen to replicate within the host interior because the host defense can only be overpowered if there is a continuous flow of pathogenic effectors within the host [26]. In the present study, SEM showed that tissue disintegration and accumulation of degraded products in the susceptible cultivar had no effect on fungal division as the pathogen was seen at different divisional stages within the xylem vessels even after 12 days of infection.

In case of the resistant cultivar, both chitinase and beta 1–3 glucanases maintained steady state levels throughout the fungal penetration process, which predicted a different function of these enzymes in disease responses in an incompatible host-pathogen encounter. Pathogenic entry was evident at later stages of infection [25 DPI] coupled with tissue disintegration, although to a comparably lower extent than that of the susceptible variety. These results suggested that in an incompatibility interaction the host somehow reprogrammed itself to obstruct pathogenic division within the host interior, thus maintaining the normal solute conduction and metabolic homeostasis within the host interior.

### Early Pathogen Recognition Responses of the Host

The host recruits its defense machinery only after it senses foreign ingress. Throughout the present study, the expression of several early pathogen recognizing genes was detected. ATPases localized in membrane organelles and plasma membrane regulate acidification by pumping protons across the plasma membrane and maintaining solute homeostasis necessary for processes like receptor-mediated endocytosis and protein sorting [27], [28]. Such acidification of intracellular compartments is reported to energize ion and metabolite transport during elicitor induced stress in soybean [29] and salt stress in *Porteresia coarctata* [30]. ATPases also acts as a possible target of Ca^++^ activated protein kinase in tomato that is induced by medium alkalinization upon pathogen invasion and wounding [31]. The activation of ATPases promotes hydrolase and transferase activities [32]. In tobacco, ATPases are considered to be a molecular switch for SA signaling and preventing JA/ET-mediated necrosis during *Pseudomonas syringae* attack [33].

Rapid alkalization factor (RALF), a polypeptide hormone, is a plant stress indicator and growth regulator causing rapid alkalinization of the growth medium. RALF was induced in the resistant cultivar *Brassica rapa* during *Plasmadiophora brassicae* infection [34]. It promotes extracellular alkalinity and activates MAP kinases in tobacco [35]. The Ser/Thr kinases act as ‘central processing units’ that accept input signals from receptors that sense external or internal stimuli (e.g., salt and carbon stress) and convert them to appropriate output signals such as changes in metabolism, gene expression, cell growth and division [36]. Osmotic stress induces Ser/Thr kinases downstream of the SA signaling pathway [37]. RALF also regulates the expression of Ser/Thr kinases [38]. In turn, Ser/Thr kinases regulate the expression of sucrose synthases and invertases during carbon stress conditions [39]. In a similar case study involving chickpea-*Fusarium* interaction such kinases were reported to be induced [17].

Phospholipase C promotes the hydrolysis of phosphoinositides into inositol triphosphate (IP_3_) and diacylglycol (DAG). DAG rapidly converts into phosphatidic acid (PA), thus promoting medium alkalinization and triggering downstream MAP kinases and calcium-dependent protein kinases [40]. Moreover, PA also activates plasma membrane ion channels probably for transmission of signals in gene for gene interactions in tomato and *Cladosporium fulvum* encounters [41].

In the present study, the transcript levels of ATPases, RALF, Ser/Thr kinase and phospholipases were found to be elevated in the resistant variety in comparison to the susceptible one from 48 h to 96 h post-infection. Hence it was presumed that the resistant variety somehow reoriented its metabolism and induced ATPases that played a crucial role in sequestering low pH fungal toxic metabolites into the vacuole and calibrating the cell for normal metabolism. RALF-mediated alkalinization probably aided ATPase expression. Phospholipase C also promoted alkalinity and RALF assisted production of Ser/Thr kinases. *Ralstonia solanacearum* induced vacuolar acidity and extracellular alkalinity coupled with Ser/Thr kinase and phospholipase C expression prior to oxidative burst in sweet potato [42]. Invertases also aided the above functions. On the whole, these early pathogen recognizing components functioned somewhat synergistically in combating the fungus. The case study of *Ralstonia solanacearum* and sweet potato supported the hypothesis except for an exception that the signaling events though entirely common to our study culminated in an oxidative burst mediated pathogen restriction that was absent in the present resistant plant-pathogen encounter, probably because an oxidative burst in the central nutrient-conducting strand could prove to be fatal for the host.

### Pathogen-Induced Wounding of Host Tissue

Fungal invasion within the host produced wounding responses evidenced by the expression of several wound-inducible genes. Arginases hydrolyse arginine to urea and ornithine, the latter being the precursor of polyamines, the well-studied wound healers. Urea gives rise to ammonia, which maintains the nitrogen pool during fungal attack [43]. Overexpression of arginases imparted resistance in tomato against *Manduca sexta* by catabolizing arginine in the insect midgut [43]. Besides, the protective role of arginase is well documented in studies where the ornithine generated via arginases helps in producing extensins at the site of wound-induced tissue damage [44]. In our case study, the enhanced expression of transcripts related to arginase in susceptible plants compared to the resistant ones suggested widespread fungal invasion within susceptible plants. However, the basal level of expression found at 48 h post-inoculation in the resistant variety also increased with increasing pathogenic invasion. Such increases in arginase related transcript expression suggested the role of the fungus in producing wounds in both varieties.

Leguminous plants produce phytoalexins and phytoanticipins prior to, during and after pathogenic attacks, and isoflavanoids form the major part [45]. Cytochrome P450 monoxygenases play important roles in isoflavanoid synthesis [46]. These cytochrome P450 monoxygenases exist as sugar conjugates inside the vacuoles and act as H_2_O_2_ scavengers [47]. Elicited licorice, soybean, pea and chickpea are the main sources of P450 monoxygenase cDNAs involved in isoflavanoid biosynthesis [48]. Pisatin demethylase, a P450 monoxygenase along with pisatin imparts resistance against *Nectria hematococca* in pea [49]. Often, sugar metabolizing genes such as sucrose synthase regulate isoflavanoid production and impart resistance as found in the case of the tobacco and *Botrytis cineria* interaction [50]. Apart from these, P450 monooxygenase catalyzes many hydroxylation reactions within plants [51]. Studies conducted on soybean showed induction of P450 monoxygenase upon elicitation with cell wall fractions of the fungal pathogen *Phytopthora megasperma* [29]. In the present study, the high expression of isoflavanoid biosynthetic genes at early hours of infection in the resistant cultivar suggests a probable role of secondary metabolites in early defense signaling that seems to be crucial for imparting resistance. The drastic reduction in expression at 96 h of induction indicated that the expression was probably not indispensable at later hours of infection for the incompatibility interaction.

Few reports regarding the stress-responsive role of DNA methylation-sensitive fragments were documented, particularly in response to cold stress in maize [52], salt stress in *Brassica* [53] and temperature and pathogen-induced changes in tobacco [54]. However, in our present study their role could not be elucidated due to a dearth of supportive literature regarding their possible functions in fungal pathogenesis.

### Pathogen-Influenced Changes in Primary Metabolism

Successful pathogens compete with the host for essential metabolites and attempt to capture its primary metabolism. On the other hand, in an incompatible reaction the host utilizes its mass energy to protect its primary metabolism from the foreign invaders. Sugar metabolism occupies a pivotal position in plant life. Plant pathogens tend to deplete sugar levels of the host, resulting in induction of sugar cleaving enzymes like sucrose synthase and invertase [55]. *F. oxysporum* f. sp. *lycopercisi* induced alterations in source-sink sugar levels in tomato along with downstream MAP kinase signaling [56]. Extracellular invertase was reported to play the key role in phloem unloading and downstream MAP kinase signaling [57]. Nitrogen fixation was influenced by sucrose synthase activity in soybean [58]. Beta amylases also participated in redox regulated starch degradation under specific stress conditions [59]. Glycoside hydrolases are involved in cell wall polysaccharide metabolism, biosynthesis and remodulation of glycans, mobilization of energy during symbiosis, signaling and stress induced secondary plant metabolism [60]. Sugar transporters play a direct role in signal transduction by regulating sugar transport during normal as well as pathogen or wound -induced stressful conditions [61], [62], [63]. In our study, the expression of ESTs sharing homology with sugar cleaving enzymes and sugar transporters emphasized the role of the fungus in inciting the host defense machinery for protecting the food-processing unit. Furthermore, the role of sugar alarms in mediating stress signals is also not surprising. Apart from this, the relatively enhanced expression of the above genes in the susceptible variety 72 h post-inoculation suggested that a similar self-protective strategy was also operational within them that probably failed to meet the extending demands at later stages of infection.

Nitrate transporters related transcripts are induced in roots as an adaptive response against nitrogen depletion in *Arabidopsis* [64]. In our study it can be assumed that fungus infection probably induced changes in nitrogen metabolism, and this was somehow compensated by the byproducts generated by arginases in resistant plants. Plastids reside both at the receiving and acting end in various cellular processes and alterations caused by environmental cues [65]. In our study, the role of upregulation of plastid division regulator related EST specifically in resistant plants was unclear. Acyl activating enzymes are induced in *Arabidopsis* in response to *Alternaria brassicicola* and *Botrytis cineria* infection [66], [67]. Also, these enzymes are related to oxylipin biosynthesis and promote intracellular acidification resulting in production of PR proteins during pathogen attack [68]. Transcripts sharing homology with such enzymes consistently showed high expression in the resistant plants in the present study. 14.3.3 regulates several protein–protein interactions during abiotic and biotic stresses [69]. They also act as receptors of fungal toxins and form a stabilized tripartite complex in association with H^+^ATPase that is responsible for leaching nutrients and resulting in wilting of plants [70]. Enhanced expression of this transcript in the susceptible cultivar suggested a probable role of fungal toxins in our present study. Moreover, the literature supports the role of 14.3.3 proteins in cleavage of their binding partners in sugar-starved cells [71]. In the present study, the overexpression of 14.3.3-like proteins in the infected susceptible variety suggests possible sugar starvation in them, while the resistant variety could make up for the shortage by overexpression of several sugar metabolizing genes.

### Induction of Structural Proteins, Transcriptional Regulators and Antifungal Components

The armadillo beta catenin repeat family proteins are transcriptional regulators that promote the structural alteration of transcription factors leading to gene activation [72]. Tubulin folding cofactor is known to regulate cell division and vesicular trafficking in *Arabidopsis* [73]. However, the significance of these genes in our study is yet to be elucidated. Ribosomal proteins like RPS6 and RPL34 are upregulated in response to wounding and abiotic stress in *Arabidopsis* [74]. They are also regulated by the sucrose, octadecanoid and lipoxygenase pathways [75]. The octadecanoid pathway along with the lipoxygenase pathway is responsible for the production of oxylipins that are important for plant defense [76]. Cytochrome C oxidase (COX), the key enzyme of aerobic respiration, is involved in the translocation of protons and has an active role in regulating stress-mediated signals [77]. The basal expression of the ribosomal protein transcripts and oxidase enzymes related ESTs in the resistant variety emphasizes their role in transcriptional regulation and signal generation probably due to pathogen-induced enhanced respiration throughout the cell during early infection. There are several reports of plant cystatins from barley, soybean, tomato and sugarcane that prevent the growth of fungal and bacterial pathogens even though their antifungal activity is not attributed to their cysteine protease activity [78], [79], [80], [81]. Hence, the role and antifungal features of cystatins related transcripts found to be upregulated in the resistant variety needs to be critically evaluated.

### Conclusion

**Figure 9:**
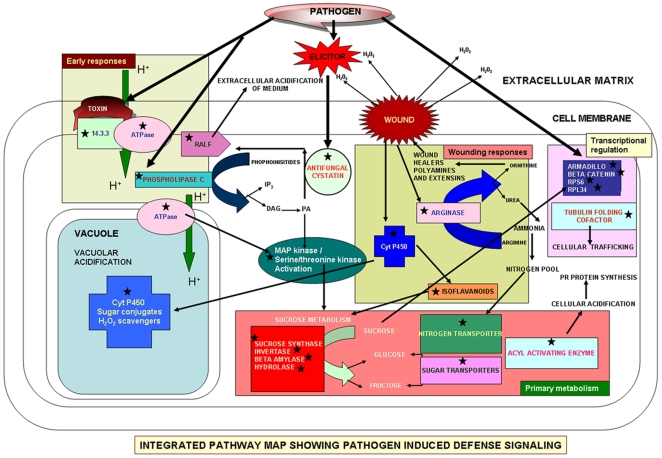
Schematic pathway predicting the role of pathogen induced genes in defense. Integrated pathway map shows the role of pathogen induced defensive genes involved in early defense, wound response, primary metabolism, transcriptional regulation and antifungal activity. The ESTs are indicated in stars.

Foc Race 1-induced changes in chickpea are summarized in a schematic pathway [Figure 9]. Experimental data suggests wound mediated entry of the pathogen within the host which was predicted by the induction of arginase, isoflavanoids, cytochrome P450 monoxygenase and DNA methylation related ESTs. The induction of ATPases, RALFs, Ser/Thr kinase and phospholipase C related ESTs signifies a somewhat early sensing of the pathogen by the host plant. Induction of all these above mentioned genes leads to altered primary metabolism of the host plant, which involves changes in sugar and nitrogen metabolism. This assumption was supported by the over expression of sugar and nitrogen metabolism related transcripts and transporters in the present study. These changes in primary metabolism may further regulate many structural and transcriptional regulators. On the whole, it is predicted that in compatible interaction Foc Race 1 establishes within the host, triggers HR, targets the host's primary metabolism and overpowers host resistance. Conversely, in resistant plants the pathogen is sensed early, its establishment within the host is delayed, HR intensity is comparably lower than the susceptible variety and host primary metabolic signals compensate for the pathogen-induced damage.

In a similar work involving chickpea and wilt causing pathogen *Fusarium oxysporum* f.sp. *ciceri* Race 1 [17] an extensive comparison between wilt related ESTs of susceptible and resistant plant varieties suggested many non-canonical genes and many unexpected candidates with known non-stress biochemical function to be involved in the immune response of chickpea. But the proper functional characterizations of such genes are still pending. Thus, further characterization of the gene clusters involved in the chickpea-*Fusarium* interaction would lead to an in-depth understanding of wilt disease management in chickpea.

## Materials and Methods

### Fungal Strain and Growth Conditions

*F. oxysporum* f.sp. *ciceris* Race 1 (Foc1) obtained from ICRISAT was purified as mentioned by Summerell et al. [82]. The harvested fungal spore suspension was stored at −80°C with 30% glycerol.

### Plant Material and Growth Conditions

Experiments were performed using chickpea (*Cicer arietinum*) seeds of two different varieties, JG62 (wilt-susceptible) and WR315 (wilt-resistant), obtained from International Crops Research Institute for the Semi-Arid Tropics (ICRISAT), Patancheru, Andhra Pradesh, India [11]. Seeds of both varieties were sown in a mixture of sand and synthetic soil taken at a ratio of 1∶1 and allowed to grow in natural greenhouse conditions suited for the crop. Seeds harvested approximately after 150–180 days of sowing were used for further experimentation.

### Fungal Bioassay

Seeds of both JG62 and WR315 were sterilized using 0.1% HgCl_2_ and germinated in autoclaved sand and synthetic soil mixture (1∶1). Twelve to fifteen-day-old seedlings of 15–20 cm were used for assays. Plants were inoculated using the sick soil treatment as described by Gupta et al. [18].

### Microscopy

For light microscopic studies serial sections of both infected and uninfected roots of JG62 and WR315 were done every 24 h after inoculation, stained with Trypan blue and Lactophenol (Himedia Laboratories, http://www.himedialabs.com) and visualized under a light microscope.

SEM experiments were performed according to the protocol documented by Thoungchaleun et al. [83]. Root portions (2 cm×2 cm) of mainly the root hair region were excised using a sharp razor blade from infected susceptible (from 2 DPI–15 DPI) and resistant (from 2 DPI–30 DPI) plants. Roots of uninoculated control plants were also sampled and processed accordingly. All the samples were fixed using 3% glutaraldehyde in 1X PBS (pH 7.2) at 4°C overnight and washed thrice with the same buffer each for 10 min. The samples were post fixed with 1% (w/v) osmium tetroxide in the same buffer at 4°C for 2 h and washed briefly with distilled water. The samples were then dehydrated in a graded ethanol series (30, 50, 70, 80, 90 and 100% each for 10 min) at room temperature. The samples were further treated with isoamyl acetate in the same graded fashion (30, 50, 70, 80, 90 and 100% each for 10 min) and dried in a critical point drier (CPD030; BALTEC, http://www.bal-tec.com) with CO_2_ as the transitional fluid. Samples were then mounted on metal stubs (10 mm in diameter) using two-sided adhesive carbon tape and coated under an argon atmosphere with a thin layer (approx. 30 nm in thickness) of gold using a sputter coater (JFC-1100E; JEOL, http://www.jeol.com) at an accelerating voltage of 20 kV.

### RNA Extraction, cDNA Preparation and cDNA-AFLP Analyses

Roots of infected and non-infected plants of both JG62 and WR315 were collected at 48 h, 72 h and 96 h post inoculation and frozen in liquid N_2_. Total RNA was extracted from the samples using a TRI reagent kit (Sigma-Aldrich, http://www.sigmaaldrich.com) according to the manufacturer's protocol. Purification of the mRNA and subsequent cDNA-AFLP analyses were performed following the method described by Gupta et al. [18]. The *Eco*RI and *Mse*I adapters and preamplification primers mentioned in Table S3 were used [84].

### Isolation, Re-Amplification and Cloning of ESTs

The differentially expressed ESTs were extracted from the AFLP gel and cloned into the pGEMT Easy vector (Promega, http://www.promega.com) according to the protocol described by Gupta et al. [18]. Sequencing of the ESTs was done on automated ABI Prism 377 Sequencer (Applied Biosystems, http://www3.appliedbiosystems.com) at the sequencing facility of Delhi University, South Campus.

### Bioinformatic Analyses of ESTs

The sequences of the ESTs (with vector sequence trimmed off, as recombinant plasmids were used as template) were analyzed for their homology against the publicly available non redundant genes/ESTs/Transcripts in the NCBI database using the BLASTN and BLASTX algorithms [85], [86], [87], [88]. The sequences were submitted to EST database of Genbank with Accession numbers listed under Table S1.

### Quantitative Real Time Polymerase Chain Reaction (qRT-PCR)

Quantitative real time PCR was performed on a BioRad iCycler (http://www.biorad.com/) using SyBr Green qPCR Supermix (2X), 25 ng of cDNA 0.3 µM of sequence specific forward and reverse primers (Table S4) in a volume of total 20 µl. PCR cycling conditions were 95°C for 5 min, followed by 40 cycles at 95°C for 30 sec, 55°C for 30 sec and 72°C for 30 sec [3]. Melt curve analyses were done to determine the primer specificities. Variations in cDNAs of the samples were normalized using actin as internal standard [89]. Fold change was calculated for 48 h, 72 h and 96 h post-inoculation in both susceptible JG62 and resistant WR315 plants. The fold changes were calculated using the 2^−δδCt^ method [90]. Experiments for the 25 genes were performed in triplicate. The average fold induction values were calculated after considering the standard error, where n = 3 (n represents the number of biological replicates, each replicate obtained by 50 individual roots pooled together).

## Supporting Information

Table S1 ESTs obtained from chickpea upon *Fusarium oxysporum* f. sp. *ciceri* (Race 1) attack by cDNA-AFLP analyses.
(0.11 MB DOC)

Table S2 Relative expression of different ESTs in chickpea generated in response to *Fusarium oxysporum* f. sp. *ciceri* (Race 1) attack using real time PCR analysis.
(0.07 MB DOC)

Table S3 Sequences of adapter, preamplification and selective amplification primers used in cDNA-AFLP analyses.
(0.09 MB DOC)

Table S4 Primer sequences used for real time PCR.
(0.05 MB DOC)
