# Supplementary material for: Primary Metabolism of Chickpea Is the Initial Target of Wound Inducing Early Sensed Fusarium oxysporum f. sp. ciceri Race I
Source: PLoS One. 2010 Feb 3;5(2):e9030. doi: 10.1371/journal.pone.0009030 (PMC2815786; doi:10.1371/journal.pone.0009030)
Supplement: Table S1 — ESTs obtained from chickpea upon Fusarium oxysporum f. sp. ciceri (Race 1) attack by cDNA-AFLP analyses. (0.11 MB DOC) [file pone.0009030.s001.doc]

**Supporting information**

**Table S1**

ESTs obtained from chickpea upon *Fusarium oxysporum* f. sp. *ciceri* (Race 1) attack by cDNA-AFLP analyses.

| EST Accession | EST size | Annotation | Type of EST | Degree of expression  WR315 | Degree of expression  JG62 |
| --- | --- | --- | --- | --- | --- |
| GO660556 | 372bp | Vacuolar proton ATPase subunit F | Early defense responsive | +++ | + |
| GO935218 | 140bp | Vacuolar proton ATPase subunit E | Early defense responsive | +++ | + |
| GO660548 | 395bp | Rapid alkalinization factor 1 precursor (RALF 1) | Early Defense responsive | +++ | + |
| GO660549 | 395bp | Rapid alkalinization factor 1 precursor  (RALF 1) | Early Defense responsive | +++ | - |
| GO660536 | 181bp | Serine threonine kinase related protein, | Early Defense  Responsive | +++ | - |
| GO660546 | 220bp | Phosphoinositide specific phospholipase C | Early Defense responsive | +++ | + |
| GO660531 | 186bp | Isoflavanoid biosynthetic gene | Wound responsive | +++ | - |
| GO660519 | 81bp | Arginase 2 | Wound responsive | - | +++ |
| GO660526 | 75bp | Arginase | Wound responsive | +++ | ++ |
| GO935220 | 75bp | Arginase | Wound responsive | +++ | ++ |
| GO660524 | 86bp | Cytochrome P450 | Wound / Stress responsive | +++ | + |
| GO660518 | 52bp | Methylation sensitive polymorohic fragment | Stress responsive | - | +++ |
| GO660535 | 190bp | Drought stress related EST | Stress responsive | +++ | + |
| GO935221 | 108bp | Beta amylase | Primary metabolism | +++ | + |
| GO660570 | 96bp | Beta amylase | Primary metabolism |  |  |
| GO660540 | 176bp | Plastid division regulator MinE | Primary metabolism | +++ | - |
| GO660561 | 133bp | Plastid division regulator | Primary metabolism | +++ | - |
| GO660552 | 141bp | Alkaline invertase | Primary metabolism | +++ | - |
| GO935217 | 118bp | Nodule enhanced sucrose synthase (ness) | Primary metabolism | +++ | - |
| GO660557 | 285bp | Hydrolase, alpha/beta fold family protein | Primary metabolism | +++ | + |
| GO660523 | 223bp | 14.3.3 like protein | Primary metabolism | - | +++ |
| GO660555 | 104bp | ATP synthase delta chain | Primary metabolism | +++ | + |
| GO660567 | 88bp | Nitrate transporter | Primary metabolism | +++ | - |
| GO660572 | 96bp | Sugar transporter | Primary metabolism | +++ | - |
| GO660573 | 68bp | Acyl activating enzyme | Primary metabolism | +++ | + |
| GO660547 | 226bp | Ubiquitin ligase | Primary metabolism | +++ | - |
| GO660551 | 144bp | Cystatin like protein | Antifungal activity | +++ | - |
| GO660545 | 220bp | Armadillo/beta catenin repeat family protein | Transcription regulation | +++ | + |
| GO660550 | 334bp | 60s ribosomal protein L34 | Transcription regulation | +++ | + |
| GO660560 | 63bp | Ribosomal protein S6 | Transcriptional regulation | +++ | + |
| Go660530 | 86bp | Cytochrome oxidase subunit 1 (COI) | Structural and signaling component | +++ | + |
| GO660520 | 100bp | Tubulin folding cofacter E | Structural | + | +++ |
| GO660525 | 86bp | Kinesin heavy hain member2 (KIF2) | Structural | +++ | + |
| GO660527 | 75bp | Leucine rich transmembrane protein | Structural | +++ | + |
| GO660528 | 120bp | Photosystem II D1 protein (PsbA) | Structural | +++ | + |
| GO660537 | 186bp | Hypothecical protein related to hexadiploidization | Unknown function | +++ | ++ |
| GO935219 | 75bp | Male enriched Y9 marker | Unknown function | +++ | ++ |
| GO660541 | 255bp | Unknown protein | Unknown function | +++ | + |
| GO660544 | 220bp | Unknown protein | Unknown function | +++ | + |
| GO660553 | 148bp | Binding mRNA | Unknown function | +++ | + |
| GO660522 | 164bp | Clone similar to *Medicago trancatula*  chromosome 3 clone MTH2-21G21 | Clone | + | +++ |
| GO660521 | 124bp | Clone similar to *Medicago trancatula* chromosome 7 BAC clone mth2-62p5 | Clone | - | +++ |
| GO660543 | 176bp | Clone similar to *Medicago trancatula* chromosome 5 clone mte1-29c13 | Clone | +++ | + |
| GO660532 | 187bp | Clone similar to *Medicago trancatula* chromosome 5 clone mth2-6h11 | Clone | +++ | ++ |
| GO660533 | 172bp | Clone similar to *Populus tricocarpa* clone POPO18-P22 | Clone | +++ | + |
| GO660534 | 185bp | Clone similar to *Lycopersicon esculentum* clone 133854F | Clone | +++ | + |
| GO660538 | 256bp | Clone similar to *Populus trichocarpa* clone WS01211 | Clone | +++ | ++ |
| GO660539 | 255bp | Clone similar to *Medicago trancatula* clone mth2-91j8 | Clone | +++ | ++ |
| GO660542 | 257bp | Clone similar to *Hordeum vulgare* sp *vulgare* cDNA clone: Flbaf30d19 mRNA | Clone | +++ | + |
| GO660529 | 75bp | Clone similar to *Medicago trancatula* chromosome 2 BAC clone mth2-1733e20 | Clone | +++ | + |
| GO660554 | 148bp | Clone similar to Solanim lycopersicon cDNA clone LEFL1003DB09 HTC of leaf | Clone | +++ | + |
| GO660558 | 68bp | Clone similar to *Medicago trancatula* clone mth2-10a3 | Clone | +++ | + |
| GO660559 | 51bp | Clone similar to *Medicago trancatula* chromosome 5 clone mth4-19c21 | Clone | +++ | + |
| GO660562 | 283bp | Clone similar to *Medicago trancatula* clone MTH2-180D15 | Clone | +++ | ++ |
| GO660563 | 160bp | Clone similar to *Medicago trancatula* clone MTH2-172C6 | Clone | +++ | ++ |
| GO660564 | 66bp | Clone similar to Human DNA sequence of clone RP11-454L1 of chromosome 1 | Clone | +++ | + |
| GO660565 | 97bp | Clone similar to Mouse DNA sequence clone RP23-294B13 | Clone | +++ | ++ |
| GO660566 | 66bp | Clone similar to Human DNA sequence  clone RP11-694J20 | Clone | +++ | ++ |
| GO660568 | 90bp | Clone similar to Human DNA sequence  clone RP11-22P4 | Clone | +++ | + |
| GO660569 | 135bp | Clone similar to *Medicago trancatula* chromosome 7 clone mte1-1i2 | Clone | +++ | + |
