# Supplementary material for: Primary Metabolism of Chickpea Is the Initial Target of Wound Inducing Early Sensed Fusarium oxysporum f. sp. ciceri Race I
Source: PLoS One. 2010 Feb 3;5(2):e9030. doi: 10.1371/journal.pone.0009030 (PMC2815786; doi:10.1371/journal.pone.0009030)
Supplement: Table S2 — Relative expression of different ESTs in chickpea generated in response to Fusarium oxysporum f. sp. ciceri (Race 1) attack using real time PCR analysis. (0.07 MB DOC) [file pone.0009030.s002.doc]

**Supporting information**

**Table S2**

Relative expression of different ESTs in chickpea generated in response to *Fusarium oxysporum* f. sp. *ciceri* (Race 1) attack using real time PCR analysis.

| EST Accession | Type of EST | Annotation | Homologous Accession Number | Expression level (2^-ddct^)  Fold Change normalized to calibrator controls | | | | | |
| --- | --- | --- | --- | --- | --- | --- | --- | --- | --- |
|  |  |  |  | JG62 | | | WR315 | | |
|  |  |  |  | 48h | 72h | 96h | 48h | 72h | 96h |
| GO660556 | Early defense responsive | Vacuolar proton ATPase subunit F | DQ486058 | 5.895 | 8.234 | 7.214 | 9.325 | 21.752 | 24.123 |
| GO935218 | Early defense responsive | Vacuolar proton ATPase subunit E | AF282970 | 20.92 | 17.15 | 0.85 | 41.899 | 116.7 | 180.98 |
| GO660549 | Early Defense responsive | Rapid alkalinization factor 1 precursor  (RALF 1) | AY172330 | 3.70 | 1.632 | 0.89 | 14.997 | 13.177 | 8.339 |
| GO660536 | Early Defense  Responsive | Serine threonine kinase related protein, | D10851 | 73.00 | 16.22 | 4.08 | 220.3 | 120.25 | 82.9 |
| GO660546 | Early Defense responsive | Phosphoinositide specific phospholipase C | BAA97337 | 9.624 | 3.881 | 2.234 | 12.979 | 32.899 | 39.034 |
| GO660531 | Wound responsive | Isoflavanoid biosynthetic gene | AP009237 | 4.03 | 8.71 | 18.50 | 88.85 | 65.49 | 2.229 |
| GO660519 | Wound responsive | Arginase 2 | AY656838 | 27.125 | 38.124 | 47.258 | 5.621 | 9.213 | 16.231 |
| GO660524 | Wound / Stress responsive | Cytochrome P450 | DQ788839 | 5.892 | 7.231 | 7.331 | 21.023 | 26.591 | 39.025 |
| GO660518 | Stress responsive | Methylation sensitive polymorohic fragment | EF061314 | 7.2356 | 17.012 | 31.020 | 5.231 | 4.1011 | 0.91 |
| GO660535 | Stress responsive | Drought stress related EST | CU229655 | 12.40 | 12.09 | 13.11 | 20.345 | 6.74 | 4.9 |
| GO935221 | Primary metabolism | Beta amylase | CA139244 | 37.185 | 119.70 | 0.68 | 104.933 | 124.787 | 94.624 |
| GO660540 | Primary metabolism | Plastid division regulator MinE | DQ118106 | 0.122 | 0.076 | 0.080 | 14.62 | 15.889 | 24.933 |
| GO660552 | Primary metabolism | Alkaline invertase | NM111456 | 7.9631 | 4.713 | 6.86 | 36.84 | 38.675 | 62.106 |
| GO935217 | Primary metabolism | Nodule enhanced sucrose synthase (ness) | AF079851 | 5.527 | 22.943 | 3.547 | 14.757 | 12.669 | 17.347 |
| GO660557 | Primary metabolism | Hydrolase, alpha/beta fold family protein | NM119911 | 8.235 | 19.235 | 28.568 | 25.231 | 20.1246 | 23.478 |
| GO660523 | Primary metabolism | 14.3.3 like protein | AJ238682 | 77.71 | 35.835 | 50.445 | 6.334 | 1.362 | 0.156 |
| GO660567 | Primary metabolism | Nitrate transporter | AB052788 | 7.89 | 19.23 | 2.35 | 17.84 | 23.51 | 31.78 |
| GO660572 | Primary metabolism | Sugar transporter | NM180633 | 11.73 | 22.11 | 5.33 | 10.53 | 35.22 | 34.21 |
| GO660573 | Primary metabolism | Acyl activating enzyme | NM112487 | 3.95 | 9.04 | 19.74 | 35.78 | 31.23 | 38.77 |
| GO660551 | Antifungal activity | Cystatin like protein | AF283536 | 17.9 | 8.734 | 1.47 | 60.439 | 97.906 | 12.799 |
| GO660545 | Transcription regulation | Armadillo/beta catenin repeat family protein | NP566136 | 2.281 | 1.620 | 1.011 | 4.055 | 5.086 | 9.105 |
| GO660550 | Transcription regulation | 60s ribosomal protein L34 | L27107 | 11.23 | 13.39 | 10.5 | 30.56 | 38.74 | 75.78 |
| GO660560 | Transcriptional regulation | Ribosomal protein S6 | AY453394 | 5.76 | 9.78 | 17.54 | 21.59 | 24.74 | 24.04 |
| Go660530 | Structural and signaling component | Cytochrome oxidase subunit 1 (COI) | EF015412 | 3.335 | 4.101 | 4.231 | 8.561 | 6.325 | 3.335 |
| GO660520 | Structural | Tubulin folding cofacter E | AAM22962 | 15.321 | 11.234 | 7.563 | 2.563 | 1.112 | 1.056 |
