# Supplementary material for: Primary Metabolism of Chickpea Is the Initial Target of Wound Inducing Early Sensed Fusarium oxysporum f. sp. ciceri Race I
Source: PLoS One. 2010 Feb 3;5(2):e9030. doi: 10.1371/journal.pone.0009030 (PMC2815786; doi:10.1371/journal.pone.0009030)
Supplement: Table S3 — Sequences of adapter, preamplification and selective amplification primers used in cDNA-AFLP analyses. (0.09 MB DOC) [file pone.0009030.s003.doc]

**Supporting information**

**Table S3**

Sequences of adapter, preamplification and selective amplification primers used in cDNA-AFLP analyses.

| **Primer Names** | **Sequences** |
| --- | --- |
| EcoR1 Adapter primer **Forward** | 5′-CTCGTAGACTGCGTACC-3 |
| EcoR1 Adapter primer **Reverse** | 3′-CTGACGCATGGTTAA-5′ |
| Mse 1 Adapter primer **Forward** | 5′-GACGATGAGTCCTGAG-3′ |
| Mse 1 Adapter primer **Reverse** | 3′-TACTCAGGACTCAT-5′ |
| Preamplification primer **Forward** | 5’-GACTGCGTACCAATTC-3′ |
| Preamplification primer **Reverse** | 5’-GACTGCGTAC CAATTC-3′ |
| **Selective amplification Primers** |  |
| E-AT/M-AG | 5’-GACTGCGTACCAATTC**AT**-3′  5’-GACTGCGTAC CAATTC**AG**-3′ |
| E-AT/M-AC | 5’-GACTGCGTACCAATTC**AT**-3′  5’-GACTGCGTAC CAATTC**AC**-3′ |
| E-AT/M-AT | 5’-GACTGCGTACCAATTC**AT**-3′  5’-GACTGCGTAC CAATTC**AT**-3′ |
| E-AAC/M-CAC | 5’-GACTGCGTACCAATTC**AAC**-3′  5’-GACTGCGTAC CAATTC**CAC**-3′ |
| E-AAC/M-CAG | 5’-GACTGCGTACCAATTC**AAC**-3′  5’-GACTGCGTAC CAATTC**CAG**-3′ |
| E-AAC/M-CAT | 5’-GACTGCGTACCAATTC**AAC**-3′  5’-GACTGCGTAC CAATTC**CAT**-3′ |
| E-AT/M-GA | 5’-GACTGCGTACCAATTC**AT**-3′  5’-GACTGCGTAC CAATTC**GA**-3′ |
| E-AT/M-GC | 5’-GACTGCGTACCAATTC**AT**-3′  5’-GACTGCGTAC CAATTC**GC**-3′ |
| E-AT/M-GT | 5’-GACTGCGTACCAATTC**AT**-3′  5’-GACTGCGTAC CAATTC**GT**-3′ |
| E-AAC/M-CTA | 5’-GACTGCGTACCAATTC**AAC**-3′  5’-GACTGCGTAC CAATTC**CTA**-3′ |
| E-AAC/M-CTC | 5’-GACTGCGTACCAATTC**AAC**-3′  5’-GACTGCGTAC CAATTC**CTC**-3′ |
| E-AAC/M-CTG | 5’-GACTGCGTACCAATTC**AAC**-3′  5’-GACTGCGTAC CAATTC**CTG**-3′ |
| E-AAC/M-CTT | 5’-GACTGCGTACCAATTC**AAC**-3′  5’-GACTGCGTAC CAATTC**CTT**-3′ |
| E-AT/M-TA | 5’-GACTGCGTACCAATTC**AT**-3′  5’-GACTGCGTAC CAATTC**TA**-3′ |
| E-AT/M-TC | 5’-GACTGCGTACCAATTC**AT**-3′  5’-GACTGCGTAC CAATTC**TC**-3′ |
| E-AT/M-TG | 5’-GACTGCGTACCAATTC**AT**-3′  5’-GACTGCGTAC CAATTC**TG**-3′ |
| E-CA/M-AC | 5’-GACTGCGTACCAATTC**CA**-3′  5’-GACTGCGTAC CAATTC**AC**-3′ |
| E-CA/M-AG | 5’-GACTGCGTACCAATTC**CA**-3′  5’-GACTGCGTAC CAATTC**AG**-3′ |
| E-CA/M-AT | 5’-GACTGCGTACCAATTC**CA**-3′  5’-GACTGCGTAC CAATTC**AT**-3′ |
| E-CA/M-GA | 5’-GACTGCGTACCAATTC**CA**-3′  5’-GACTGCGTAC CAATTC**GA**-3′ |
| E-CA/M-GC | 5’-GACTGCGTACCAATTC**CA**-3′  5’-GACTGCGTAC CAATTC**GC**-3′ |
| E-AAC/M-CT | 5’-GACTGCGTACCAATTC**AAC**-3′  5’-GACTGCGTAC CAATTC**CT**-3′ |
| E-AAC/M-CG | 5’-GACTGCGTACCAATTC**AAC**-3′  5’-GACTGCGTAC CAATTC**CG**-3′ |
| E-AAC/M-CA | 5’-GACTGCGTACCAATTC**AAC**-3′  5’-GACTGCGTAC CAATTC**CA**-3′ |
| E-CA/M-GT | 5’-GACTGCGTACCAATTC**CA**-3′  5’-GACTGCGTAC CAATTC**GT**-3′ |
| E-AAG/M-CAA | 5’-GACTGCGTACCAATTC**AAG**-3′  5’-GACTGCGTAC CAATTC**CAA**-3′ |
| E-AAG/M-CAC | 5’-GACTGCGTACCAATTC**AAG**-3′  5’-GACTGCGTAC CAATTC**CAC**-3′ |
| E-AAG/M-CAT | 5’-GACTGCGTACCAATTC**AAG**-3′  5’-GACTGCGTAC CAATTC**CAT**-3′ |
| E-ACA/M-CAA | 5’-GACTGCGTACCAATTC**ACA**-3′  5’-GACTGCGTAC CAATTC**CAA**-3′ |
| E-ACA/M-CAC | 5’-GACTGCGTACCAATTC**ACA**-3′  5’-GACTGCGTAC CAATTC**CAC**-3′ |
| E-ACA/M-CAG | 5’-GACTGCGTACCAATTC**ACA**-3′  5’-GACTGCGTAC CAATTC**CAG**-3′ |
| E-ACA/M-CAT | 5’-GACTGCGTACCAATTC**ACA**-3′  5’-GACTGCGTAC CAATTC**CAT**-3′ |
| E-ACA/M-CTA | 5’-GACTGCGTACCAATTC**ACA**-3′  5’-GACTGCGTAC CAATTC**CTA**-3′ |
| E-AAG/M-CAG | 5’-GACTGCGTACCAATTC**AAG**-3′  5’-GACTGCGTAC CAATTC**CAG**-3′ |
| E-AAG/M-CTA | 5’-GACTGCGTACCAATTC**AAG**-3′  5’-GACTGCGTAC CAATTC**CTA**-3′ |
| E-AAG/M-CTC | 5’-GACTGCGTACCAATTC**AAG**-3′  5’-GACTGCGTAC CAATTC**CTC**-3′ |
| E-ACC/M-CAA | 5’-GACTGCGTACCAATTC**ACC**-3′  5’-GACTGCGTAC CAATTC**CAA**-3′ |
| E-ACC/M-CAC | 5’-GACTGCGTACCAATTC**ACC**-3′  5’-GACTGCGTAC CAATTC**CAA**-3′ |
| E-ACC/M-CAT | 5’-GACTGCGTACCAATTC**ACC**-3′  5’-GACTGCGTAC CAATTC**CAT**-3′ |
| E-ACC/M-CAG | 5’-GACTGCGTACCAATTC**ACC**-3′  5’-GACTGCGTAC CAATTC**CAG**-3′ |
| E-ACC/M-CTA | 5’-GACTGCGTACCAATTC**ACC**-3′  5’-GACTGCGTAC CAATTC**CTA**-3′ |
| E-ACC/M-CTC | 5’-GACTGCGTACCAATTC**ACC**-3′  5’-GACTGCGTAC CAATTC**CTC**-3′ |
| E-ACG/M-CAA | 5’-GACTGCGTACCAATTC**ACG**-3′  5’-GACTGCGTAC CAATTC**CAA**-3′ |
| E-ACG/M-CAC | 5’-GACTGCGTACCAATTC**ACG**-3′  5’-GACTGCGTAC CAATTC**CAC**-3′ |
| E-ACG/M-CAT | 5’-GACTGCGTACCAATTC**ACG**-3′  5’-GACTGCGTAC CAATTC**CAT**-3′ |
| E-ACG/M-CAG | 5’-GACTGCGTACCAATTC**ACG**-3′  5’-GACTGCGTAC CAATTC**CAG**-3′ |
| E-ACG/M-CTA | 5’-GACTGCGTACCAATTC**ACG**-3′  5’-GACTGCGTAC CAATTC**CTA**-3′ |
| E-ACG/M-CTC | 5’-GACTGCGTACCAATTC**ACG**-3′  5’-GACTGCGTAC CAATTC**CTC**-3′ |
| E-ACT/M-CAA | 5’-GACTGCGTACCAATTC**ACT**-3′  5’-GACTGCGTAC CAATTC**CAA**-3′ |
| E-ACT/M-CAC | 5’-GACTGCGTACCAATTC**ACT**-3′  5’-GACTGCGTAC CAATTC**CAC**-3′ |
| E-ACT/M-CAT | 5’-GACTGCGTACCAATTC**ACT**-3′  5’-GACTGCGTAC CAATTC**CAT**-3′ |
| E-ACT/M-CTA | 5’-GACTGCGTACCAATTC**ACT**-3′  5’-GACTGCGTAC CAATTC**CTA**-3′ |
| E-ACT/M-CTC | 5’-GACTGCGTACCAATTC**ACT**-3′  5’-GACTGCGTAC CAATTC**CTC**-3′ |
| E-AGC/M-CAA | 5’-GACTGCGTACCAATTC**AGC**-3′  5’-GACTGCGTAC CAATTC**CAA**-3′ |
| E-AGC/M-CAC | 5’-GACTGCGTACCAATTC**AGC**-3′  5’-GACTGCGTAC CAATTC**CAC**-3′ |
| E-AGC/M-CAT | 5’-GACTGCGTACCAATTC**AGC**-3′  5’-GACTGCGTAC CAATTC**CAT**-3′ |
| E-AGC/M-CAG | 5’-GACTGCGTACCAATTC**AGC**-3′  5’-GACTGCGTAC CAATTC**CAG**-3′ |
| E-AGC/M-CTA | 5’-GACTGCGTACCAATTC**AGC**-3′  5’-GACTGCGTAC CAATTC**CTA**-3′ |
| E-AGC/M-CTC | 5’-GACTGCGTACCAATTC**AGC**-3′  5’-GACTGCGTAC CAATTC**CTC**-3′ |
| E-AGG/M-CAA | 5’-GACTGCGTACCAATTC**AGG**-3′  5’-GACTGCGTAC CAATTC**CAA**-3′ |
| E-AGG/M-CAC | 5’-GACTGCGTACCAATTC**AGG**-3′  5’-GACTGCGTAC CAATTC**CAC**-3′ |
| E-AGG/M-CAG | 5’-GACTGCGTACCAATTC**AGG**-3′  5’-GACTGCGTAC CAATTC**CAG**-3′ |
| E-AGG/M-CAT | 5’-GACTGCGTACCAATTC**AGG**-3′  5’-GACTGCGTAC CAATTC**CAT**-3′ |
| E-AAC/M-CAA | 5’-GACTGCGTACCAATTC**AAC**-3′  5’-GACTGCGTAC CAATTC**CAA**-3  ′ |
| E-AAG/M-CTG | 5’-GACTGCGTACCAATTC**AAG**-3′  5’-GACTGCGTAC CAATTC**CTG**-3′ |
| E-AAG/M-CTT | 5’-GACTGCGTACCAATTC**AAG**-3′  5’-GACTGCGTAC CAATTC**CTT**-3′ |
