# Supplementary material for: Primary Metabolism of Chickpea Is the Initial Target of Wound Inducing Early Sensed Fusarium oxysporum f. sp. ciceri Race I
Source: PLoS One. 2010 Feb 3;5(2):e9030. doi: 10.1371/journal.pone.0009030 (PMC2815786; doi:10.1371/journal.pone.0009030)
Supplement: Table S4 — Primer sequences used for real time PCR. (0.05 MB DOC) [file pone.0009030.s004.doc]

**Supporting information**

**Table S4**

Primer sequences used for real time PCR.

| EST Accession | Annotation | Sequence |
| --- | --- | --- |
| GO660556 | Vacuolar proton ATPase subunit F | Forward: 5’ TGT ACA TTA TGG CCA ATA ATC 3’  Reverse: 5’ CAT CTT GAA GAT CTA TCA CAG 3’ |
| GO935218 | Vacuolar proton ATPase subunit E | Forward: 5’ GAT AAG AAG AAG ATC CGT CAA G 3’  Reverse: 5’ GAT TGA TGA GTC CTG AGT AAC 3’ |
| GO660549 | Rapid alkalinization factor 1 precursor  (RALF 1) | Forward: 5’ CAG TTA TAG TAA GAA GCG CCA C 5’  Reverse: 5’ CAC AGA GAT CAA CAG GCG TAT 3’ |
| GO660536 | Serine threonine kinase related protein, | Forward: 5’ CAA TTG GAC CAC ACA TGA AC 3’  Reverse: 5’ CAG TTA GAC CAG ACA TGA AC 3’ |
| GO660546 | Phosphoinositide specific phospholipase C | Forward: 5’ CCA AAC CGC TGA AAG AAG TGC 3’  Reverse: 5’ CAC ATC ATC GCT GTT ATC CAC 3’ |
| GO660531 | Isoflavanoid biosynthetic gene | Forward: 5’ GAC GAA GAG CGC AAT GAG 3’  Reverse: 5’ CAC AAT ACA TCA GTT AGA C 3’ |
| GO660519 | Arginase 2 | Forward: 5’ GAC TGC GTA CCA ATT CAA CG 3’  Reverse: 5’ GAG TCC TGA GTA ACA TAC TG 3’ |
| GO660524 | Cytochrome P450 | Forward: 5’ CAA CAT GAT TTC ATT GTG AG 3’  Reverse: 5’ GAG TCC TGA GTA ACT CTC 3’ |
| GO660518 | Methylation sensitive polymorohic fragment | Forward: 5’ GAC TGC GTA CCA ATT C 3’  Reverse: 5’ GAT GAG TCC TGA GTA AC 3’ |
| GO660535 | Drought stress related EST | Forward: 5’ CAG TTT GAC CAC ACA TGA AC 3’  Reverse: 5’ CAG ACA TGA ATG AAA AGC AC 3’ |
| GO935221 | Beta amylase | Forward: 5’ GCT ATT GGT AAA TAA CCA TC 3’  Reverse: 5’ CTA GTG ATT GAT GAG TCC TGA G 3’ |
| GO660540 | Plastid division regulator MinE | Forward: 5’ GCA AGC CAG TTT TTG CAG TCC 3’  Reverse: 5’ GAG TCC TGA GTA ACA GCA TC 3’ |
| GO660552 | Alkaline invertase | Forward: 5’ GAG ATC CAA GCT TTG TTC 3’  Reverse: 5’ GAG TAA CAT CTA CTG ATC 3’ |
| GO935217 | Nodule enhanced sucrose synthase (ness) | Forward: 5’ GGA GAT CAG CGA CGC GTT CG 3’  Reverse: 5’ GAG TCC TGA GTA ACT GGT G 3’ |
| GO660557 | Hydrolase, alpha/beta fold family protein | Forward: 5’ GAC TTA GCT GTA CCA GTC GTT G 3’  Reverse: 5’ GAG TAT CAC CGG AAT GAC AAC 3’ |
| GO660523 | 14.3.3 like protein | Forward: 5’ CAA TAC ATC AGT TAG ACC 3’  Reverse: 5’ CTC CAG AAT AAG GAA CAC 3’ |
| GO660567 | Nitrate transporter | Forward: 5’ GAC TGC GTA CCA ATT CAA CCG 3’  Reverse: 5’ GAT GAG TCC TGA GTA ACT G 3’ |
| GO660572 | Sugar transporter | Forward: 5’ GTC TCA GGA ACT AGT TTT G 3’  Reverse: 5’ CCT TAT GAC TTG GAG TTC 3’ |
| GO660573 | Acyl activating enzyme | Forward: 5’ CCG TGG AAC CTG CGT AGA GAG 3’  Reverse: 5’ CTT ATA TGA AGT ACA TGT G 3’ |
| GO660551 | Cystatin like protein | Forward: 5’ CGG AGT ACA ACA AAC AGT CAG 3’  Reverse: 5’ GAG ACG GTA GTT TGT TCC G 3’ |
| GO660545 | Armadillo/beta catenin repeat family protein | Forward: 5’ CGC TAG TGG CTC TGT CGC A 3’  Reverse: 5’ CAC CAC CAT TAC TAA TGG 3’ |
| GO660550 | 60s ribosomal protein L34 | Forward: 5’ GTG AGC CGT GCA TAT GG 3’  Reverse: 5’ GTC TGC TTT TCC TTT GAC 3’ |
| GO660560 | Ribosomal protein S6 | Forward: 5’GGT TAT GTC TTC AAA ATC 3’  Reverse: 5’ CAC TCC CTG CTT CAT TGG 3’ |
| Go660530 | Cytochrome oxidase subunit 1 (COI) | Forward: 5’ CAC ATT TGC CAA ATT GTG AGT 3’  Reverse: 5’ GAG TCC TGA GTAACT CTC AT 3’ |
| GO660520 | Tubulin folding cofacter E | Forward: 5’ GAC TGC GTA CCA ACG AGT C 3’  Reverse: 5’ GAT GAG TCC TGA GTG TAT TTC 3’ |
